# The Brain, the Eating Plate, and the Gut Microbiome: Partners in Migraine Pathogenesis

**DOI:** 10.3390/nu16142222

**Published:** 2024-07-11

**Authors:** Parisa Gazerani, Laura Papetti, Turgay Dalkara, Calli Leighann Cook, Caitlin Webster, Jinbing Bai

**Affiliations:** 1 Department of Life Sciences and Health, Faculty of Health Sciences, Oslo Metropolitan University, 0130 Oslo, Norway; 2 Department of Health Science & Technology, Faculty of Medicine, Aalborg University, 9260 Gistrup, Denmark; 3 Developmental Neurology, Bambino Gesù Children’s Hospital, IRCCS, Piazza di Sant’Onofrio 4, 00165 Rome, Italy; laura.papetti@opbg.net; 4 Departments of Neuroscience and Molecular Biology and Genetics, Bilkent University, Ankara 06800, Turkey; t.dalkara@bilkent.edu.tr; 5 Emory Brain Health Center, General Neurology, Atlanta, GA 30329, USA; calli.cook@emoryhealthcare.org; 6 Nell Hodgson Woodruff School of Nursing, Emory University, Atlanta, GA 30322, USA; caitlin.webster@emory.edu (C.W.); jinbing.bai@emory.edu (J.B.); 7 Winship Cancer Institute, Emory University, Atlanta, GA 30322, USA

**Keywords:** migraine, diet, gut health, microbiome–gut–brain axis, symptoms

## Abstract

This review summarizes the relationship between diet, the gut microbiome, and migraine. Key findings reveal that certain dietary factors, such as caffeine and alcohol, can trigger migraine, while nutrients like magnesium and riboflavin may help alleviate migraine symptoms. The gut microbiome, through its influence on neuroinflammation (e.g., vagus nerve and cytokines), gut–brain signaling (e.g., gamma-aminobutyric acid), and metabolic function (e.g., short-chain fatty acids), plays a crucial role in migraine susceptibility. Migraine can also alter eating behaviors, leading to poor nutritional choices and further exacerbating the condition. Individual variability in diet and microbiome composition highlights the need for personalized dietary and prebiotic interventions. Epidemiological and clinical data support the effectiveness of tailored nutritional approaches, such as elimination diets and the inclusion of beneficial nutrients, in managing migraine. More work is needed to confirm the role of prebiotics, probiotics, and potentially fecal microbiome translation in the management of migraine. Future research should focus on large-scale studies to elucidate the underlying mechanisms of bidirectional interaction between diet and migraine and develop evidence-based clinical guidelines. Integrating dietary management, gut health optimization, and lifestyle modifications can potentially offer a holistic approach to reducing migraine frequency and severity, ultimately improving patient outcomes and quality of life.

## 1. Introduction

Migraine is a prevalent and debilitating neurological disorder characterized by recurrent episodes of severe headache [1], often accompanied by nausea, vomiting, and higher sensitivity to light and sound [2]. Affecting both children and adults, migraine represents a significant public health challenge due to its impact on quality of life and productivity [3,4]. Emerging evidence suggests that migraine is not solely a neurological condition but also involves complex metabolic processes [5,6]. This review focuses on the contribution of metabolic, microbiome–gut–brain (MGB) axis, and dietary aspects in the multifaceted nature of migraine, emphasizing environmental and lifestyle factors.

The significance of exploring the interplay between the brain, eating habits, and the gut microbiome in migraine pathogenesis cannot be overstated [7]. Recent research indicates that dietary habits and gut health may play crucial roles in the onset and severity of migraine attacks [8]. The MGB axis is an active field of investigation that offers new insights into how dietary components and the gut microbiome can influence migraine [9,10].

The purpose of this review is to present current findings on the relationship between diet, the gut microbiome, and migraine, providing an overview of how these factors interact to influence migraine development and severity. By integrating evidence, this review aims to highlight potential targets and lifestyle modifications that could mitigate migraine frequency and severity, ultimately contributing to more effective management strategies for this complex disorder. The viewpoint here also considers the bidirectional relationship between migraine and dietary choices and their consequences.

## 2. Trigeminovascular System and Migraine Pathogenesis

There is consensus that the activation followed by sensitization of meningeal nociceptors within trigeminal ophthalmic and upper cervical nerves generates headaches in both migraine with and without aura [11]. Consistent with this anatomical arrangement, meningeal nociception refers to pain in the skin of the forehead and occipital regions, a pattern typical for visceral pain (e.g., heartache). Animal studies demonstrate that prolonged activation of trigeminocervical nociceptors prompts the release of vasoactive mediators such as calcitonin gene-related peptide (CGRP), pituitary adenylate cyclase-activating polypeptide (PACAP), substance P, and neurokinin-A from their nerve endings [12,13]. These peptides initiate a sterile neurogenic inflammation characterized by meningeal vasodilatation, plasma protein extravasation, the activation of meningeal inflammatory dendritic cells and macrophages, and the degranulation of dural mast cells [12,14]. A connection in the brain stem between the second-order central nociceptive neurons located in the trigeminal nucleus caudalis (TNC) and the parasympathetic superior salivary nucleus causes the release of acetylcholine, nitric oxide, vasointestinal peptide (VIP), and PACAP from parasympathetic fibers around dural blood vessels [11,15]. This trigeminoparasympathetic reflex contributes to meningeal vasodilation induced by the peptides released from nociceptors [16,17]. Of note, vascular dilation and permeability increase do not directly cause headaches, but the sensitization of Aδ fibers triggered by CGRP seems to play a primary role [12,18]. Inflammatory mediators such as prostaglandins, histamine, and tryptase released from mast cells located near dural sensory fibers can also contribute to nociceptor activation and sensitization [19]. Sensitized perivascular nociceptors responding to mechanical stimuli start firing with vascular pulsations or head motion, giving a headache a throbbing nature and leading to sensations like “my heart is beating inside my head” or “my brain is being pushed out of my eyeballs” [20]. Clinical success with largely blood–brain barrier (BBB)-impermeable triptans, which inhibit CGRP release, and the anti-CGRP antibodies suggest that CGRP has a more prominent role in meningeal neurogenic inflammation in humans [21,22]. This also supports the idea that migraine headache is initiated in the meninges because central nociceptive pathways in CNS are located behind the BBB.

The cell bodies of C-type unmyelinated and lightly myelinated A∂-type fibers reside in the trigeminal ganglion (TG) [23]. CGRP is released from activated C-type nociceptors and binds to its receptors on neighboring Aδ neurons that do not express CGRP [24,25]. This interaction may lead to signal amplification within the TG. Additionally, CGRP stimulates the satellite glia cells in the ganglion, triggering the release of nitric oxide, which in turn creates a positive feedback amplification loop by increasing CGRP release from C-type fibers [25,26]. Similar to the meninges, the trigeminal ganglion is accessible by triptans and anti-CGRP antibodies due to the lack of a BBB. The central processes of trigeminal and cervical nociceptive neurons enter the CNS and synapse on the second-order sensory neurons in the TNC extending from the upper cervical medulla spinalis segments to the medulla oblongata. Central terminals of C-fibers also release CGRP, as do their meningeal nerve endings. CGRP stimulates its receptors on TNC neurons as well as on the presynaptic terminals of A∂-fibers, promoting glutamate release [27]. Consequently, activity in the first-order TG neurons is transmitted to the second-order neurons, priming them to be excited with lower-intensity synaptic inputs than normal [20,28]. The sensitization of the TNC results in allodynia, the painful perception of non-nociceptive stimuli like touching the head [28]. TNC activity is further transmitted to the ventral posteromedial thalamic nucleus, somatosensory cortex, insula, limbic structures, and hypothalamus, leading to pain perception, as well as emotional (like mood changes), hypothalamic (like malaise), and autonomic symptoms (like pale skin) [28]. Over time, thalamic synapses may also sensitize, resulting in whole-body (extracephalic) allodynia such as the noxious perception of the touch of bracelets on the wrist. The convergence of nociceptive and visual stimuli in some thalamic neurons likely underlies photophobia, another typical migraine symptom [28,29].

The mechanisms that episodically activate the trigeminocervicovascular system in migraine within an otherwise healthy brain are not entirely clear. The occurrence of migraine prodrome often preceding the headache suggests that the loss of homeostasis begins intrinsically in the brain parenchyma in most cases. Consistent with this perspective, prodromal symptoms are anatomically associated with cortical and subcortical structures [30]. Experimental animal studies support the notion that cortical spreading depolarization (CSD), the putative cause of migraine aura, can induce headache by initiating a parenchymal inflammatory signaling cascade that progresses to the meninges by way of astrocytes [31,32]. Notably, mutations identified in familial migraine increase susceptibility to CSD in animal models [33]. Furthermore, clinical investigations using a highly sensitive PET ligand have revealed an inflammatory process encompassing both the cerebral parenchyma and adjacent meninges in patients experiencing frequent migraine with aura attacks [34,35].

However, there are distinct examples that seem to involve mainly meningeal activation without a primary parenchymal component, thus triggering migraine headaches. For instance, the Umbellularia California plant, known as the “headache tree”, can induce a migraine episode in susceptible individuals through inhalation of the volatile compound umbellullone emitted by its leaves. This is believed to occur via direct activation of meningeal nociceptive afferents by opening TRPA1 channels on nociceptors, subsequently leading to CGRP release [36]. Some volatile irritants known to trigger migraine headaches may also act through a similar mechanism. Additionally, GTN or CGRP infusion has been suggested to trigger migraine attacks in migraineurs, likely through peripheral mechanisms [22,37]. Allergy-triggered migraine attacks may involve mast cell degranulation and subsequent dural inflammation [38]. The throbbing headache accompanying fever during systemic infections may also result from the activation and sensitization of meningeal nociceptors by circulating pro-inflammatory cytokines. The absence of the BBB at the level of dural nociceptors, as well as at the trigeminal and dorsal root ganglia, provides a common platform for several circulating pro-inflammatory mediators and molecules to trigger migraine headaches. Similar genetic predispositions (e.g., TRES potassium channel mutations [39]) and hormonal factors (e.g., estrogen [38]) can prime both parenchymal and meningeal mechanisms for migraine attacks, with parenchymal disturbances likely associated with auras and prodromal symptoms. Additionally, synaptic metabolic stress caused by transcriptional changes induced by hunger or lack of sleep has been proposed to initiate parenchymal inflammatory signaling resulting in headache [40,41]. Although relatively underexplored, migraine triggers may also modulate nociception and dural neurogenic inflammation, predisposing people to headache generation, as proposed for estrogen and stress [38].

## 3. Microbiome Contribution to Migraine

### 3.1. Overview of the Microbiome–Gut–Brain Axis

As more attention has been paid to the human microbiome since the initiation of the Human Microbiome Project in 2007 [42,43], the microbiome–gut–brain (MGB) axis, a bidirectional communication network between the gut and the brain [44], has been a central focus in understanding the mechanisms of neurocognitive and psychological effects of gut–brain communication. As a complex communication network, the MGB axis includes the gastrointestinal (GI)-related immune system, enteric neuroendocrine system, enteric nervous system (ENS), central nervous system (CNS), and the gut microbiome [45]. The gut microbiome is defined as the collection of all genomes of microbes (e.g., bacteria, fungi, and viruses) and their functional products (e.g., metabolites) in the GI tract [46]. The human gut hosts tens of trillions of microbial cells, indicating more than 500 bacterial species on average [47]. The community of the gut microbiome plays an important role in human health and disease [48]. For example, the bidirectional MGB axis was significantly linked with GI-associated disorders, such as inflammatory bowel disease (IBD) [49] and irritable bowel syndromes (IBS) [50,51]. Recently, the MGB axis was identified to play a critical role in a wide range of neuropsychiatric disorders, including emotional disorders (e.g., acute and chronic stress and depression), developmental disorders (e.g., autism spectrum disorder), and neurodegenerative disorders (e.g., Parkinson’s disease and Alzheimer’s disease) [52,53,54].

Rapidly evolving sequencing methods and analytical tools promote our understanding of the gut microbiome and its function in human health and diseases [55]. A dysbiotic gut microbiome (i.e., loss of keystone taxa, loss of diversity, shifts in metabolic capacity, or blooms of pathogens [56,57]) has been characterized in various diseases and conditions [48,58,59]. Specifically, a dysbiotic gut microbiome could influence the brain and neurological health through multiple pathways: regulating the production of pro-inflammatory cytokines and immune activity; adjusting the production of neurotransmitters (e.g., gamma-aminobutyric acid [GABA] and serotonin); modulating the formation of bacterial metabolites (e.g., short-chain fatty acids [SCFAs]) and tryptophan/kynurenine pathway metabolites (e.g., indoles); and disturbing the neuronal pathways (e.g., afferent vagus nerve and spinal sensory neurons) [60,61]. The systems biological view of the MGB axis provides an innovative and dynamic model to study the pathophysiology of brain and neuropsychiatric disorders, in which pathophysiological processes were previously hypothesized to be limited to the brain. The MGB axis has opened newer opportunities to target the gut microbiome as a treatment for both GI disorders (e.g., IBD and IBS) and neuropsychiatric disorders (e.g., depression, Alzheimer’s disease, Parkinson’s disease, and autism spectrum disorder).

Although the connection between the GI tract and the CNS has been hypothesized as a mechanism for a wide range of human diseases and conditions, recent work proposes that the axis serves as a highway—enabling bidirectional signals that can influence peripheral and central pain [62,63,64]. In the CNS, the gut microbiome-derived or activated mediators regulate neuroinflammation via the activation of microglia, astrocytes, and immune cells to modulate the central sensitization, subsequently being linked with chronic pain [62,65,66] and pain attacks (e.g., migraine) [62,67]. Additionally, the gut microbiome-derived signaling molecules, such as metabolites, neurotransmitters, and neuromodulators, serve as critical regulators for peripheral sensitization, directly or indirectly regulating the excitability of primary nociceptive neurons and pain experiences [63,68]. Considering that the gut microbiome may regulate pain in the peripheral and central nervous system, targeting the gut microbiome by pharmacological and non-pharmacological (e.g., diet) interventions may represent a new therapeutic strategy for the management of chronic pain and other chronic diseases or conditions. Regarding migraine, studies suggest that certain foods may be migraine triggers and promote these painful events. However, food triggers do not create a clear linear reaction and can be challenging for patients and clinicians to identify. In addition to potential food triggers, patients with migraine have disproportional GI symptoms, including nausea, vomiting, and bowel disturbances prior to and throughout the migraine episode as compared to healthy controls without migraine [69]. A recent study [7] suggests that patients with gut dysbiosis are more likely to report migraine symptoms. This evidence suggests an area of further investigation to determine the best treatment approaches to migraine.

### 3.2. Role of the Gut Microbiome in Neurological Function and Dysfunction and Potential Mechanisms

Both animal and human studies indicate that the gut microbiome plays a role in maintaining homeostasis and regulating almost every major body system, including the CNS [52,70]. According to the MGB axis as mentioned above, biological pathways through which the gut influences neurological function (i.e., the brain), or vice versa, include altered gut microbiome composition and its functional metabolome, lack of balance of “beneficial” and “detrimental” bacteria in the lumen, and activated neuro-immune signaling pathways [71]. Specifically, associations between the gut microbiome and neurological function have been primarily conducted in animal models [72,73,74,75,76]. Germ-free mice (i.e., those lacking the gut microbiome) showed exaggerated responses in inflammatory pain [74], anxiety [76,77,78], and cognitive dysfunction as compared with specific pathogen-free mice [76]. However, mice infected with pathogenic *Citrobacter rodentium* received daily probiotics (*Lactobacillus rhamnosus* + *Lactobacillus helveticus*) and then showed a reversed memory dysfunction [76]. Although limited, a similar role of the gut microbiome in neurological function has been reported among human studies, including the effects of the gut microbiome on pain, anxiety, depression, and cognitive dysfunction [52,70]. Additionally, the metabolites produced or activated by the gut microbiome, such as SCFAs [79,80] and tryptophan (for kynurenine pathway metabolism) [81], may influence the relationship between the gut microbiome and neurological function [82]. Alterations in SCFAs might underpin disturbances in the CNS from neurodevelopmental disorders (e.g., mood disorders) [83]. Similarly, tryptophan metabolism via the kynurenine pathway regulates neuronal function [84] and can modulate various neuropsychiatric disorders, including depression and cognitive dysfunction [85,86,87,88].

Considering the potentially similar biological pathways underlying neurological disorders, multiple intensive reviews [52,70,89] have reviewed and summarized the role of the gut microbiome in brain function in the context of neurological disorders. Particularly, the MGB axis has been implicated in understanding the pathophysiology of a group of neurological disorders, including autism spectrum disorder, Alzheimer’s disease, multiple sclerosis, and Parkinson’s disease [52,70,89,90]. Common themes have emerged from these studies, including (1) alterations in the composition of the microbiome [91,92,93,94]; (2) involvement of the MGB axis pathways, such as α-synuclein signaling to the brain via the vagus nerve [95,96,97], pro-inflammatory cytokines and neuroinflammation [98,99,100], and functional metabolomic molecules (e.g., SCFAs) [101,102,103]; and (3) prebiotic (e.g., galacto-oligosaccharide) and probiotic (e.g., *Lactobacillus acidophilus*, *Lactobacillus casei*, *Bifidobacterium bifidum*, and *Lactobacillus fermentum*) interventions with promising results to improve GI and brain-related symptoms and behaviors [104,105,106,107]. Considering the limitations of these studies, such as primarily cross-sectional designs, participant selection bias, inconsistent sampling, and sequencing protocols [70], the findings of the MGB axis in neurological disorders need to be cautiously interpreted. However, these studies provide promising examples to further uncover the role of the MGB axis in neurological disorders, including migraine.

### 3.3. Microbiome–Gut–Brain Axis in Migraine Pathogenesis

Migraine is a complex neurological disorder that involves both the sensory afferent nerves in the face, head, and neck, intra- and extracranial vessels, and central brain structures. Migraine was once thought to be a vascular phenomenon and drugs were created to target this pathway; however, this theory is less likely to be the cause of migraine. Subsequent studies demonstrated minimal vascular change during a migraine attack [108,109]. However, the drugs that target this pathway have neural effects in addition to their vasoconstrictive effects. Triptans most likely reduce migraine symptoms through these neural effects [110].

The neural theory suggests that migraine begins in the central brain areas and if treated early, worsening migraine symptoms could be prevented. Nitric oxide (NO) can trigger migraine symptoms. NO synthase inhibition has been studied as a potential treatment option and was not an overall useful strategy as there are significant cardiovascular adverse effects associated with inhibition of NO. However, experimental migraine attacks triggered with NO are a response to triptan treatment [111]. Furthermore, migraine attacks triggered by increased levels of NO suggest a potential cause due to interplay with CGRP. Increased levels of CGRP are thought to promote migraine attacks and have been associated with higher levels of NO. This aligns with the neural theory of migraine [111]. The pathway of NO in migraine supported that nitrate-, nitrite-, and nitric oxide-reducing oral microbes (e.g., *Streptococcus* and *Pseudomonas*) play significant roles in migraine development [112].

Recently, multiple observational studies have reported the promising role of the MGB axis in migraine as well as possible targets for treatment interventions. Qu and colleagues [113] recently showed that *Lachnospiraceae UCG001* was associated with an increased risk of migraine, while *Eubacterium* and *Bacteroides fragilis* may be associated with a lower migraine risk. The functional pathways of these bacteria, including Methionine synthesis and hydrocinnamate, were associated with lower migraine risk [113]. Additionally, children with migraine showed a lower alpha diversity and dissimilar beta diversity compared to children without migraine [114]. Considering the limited literature using the observational research design primarily, future work is required to examine the role of the MGB axis in migraine among different populations.

### 3.4. Therapeutic Interventions Targeting the Gut Microbiome for Migraine Management

#### 3.4.1. Prebiotics and Probiotics

Prebiotics are found in certain foods and are the fuel for the human microbiome, whereas probiotics can be consumed either in food or supplements that contain live microorganisms or helpful bacteria. Dysbiosis has been suggested to be associated with migraine headaches [114]. The increased association between dysbiosis and migraine is thought to be related to a disproportional number of bacteria found in migraine patients’ guts that produce NO. The elevation of NO is thought to increase levels of CGRP, suggesting a potential trigger for a migraine attack [111]. Various studies, including a systematic review [115,116,117], have evaluated the usefulness of probiotics (*Bifidobacterium*, *Lactobacillus*, *Lactococcus*, and *Streptococcus*) in the prevention of migraine attacks in migraine patients. Due to methodological differences, there is no consensus on whether supplementation is helpful to migraine patients. Probiotics may have a positive influence on migraine by decreasing both the frequency and severity of attacks; however, further studies are needed to better understand the usefulness of these therapies in migraine patients [117].

#### 3.4.2. Lifestyle Interventions

Lifestyle modification is essential to migraine care and should be incorporated into the patient treatment plan. Evidence-based lifestyle modifications include SEEDS, which stands for Sleep, Exercise, Eating, Diary, and Stress Reduction [118]. Patients with migraine should aim to get seven hours of sleep per night. Patients who get poor sleep are at increased risk of migraine [119]. Consider asking patients to take 400–600 mg of magnesium prior to bed. Magnesium can prevent migraine and can help patients relax and initiate sleep. Magnesium is also thought to have a positive effect on the microbiome [7]. Patients with migraine should aim for 150 min of exercise per week or 30 min of exercise five times per week. There is no specific recommendation regarding what type of exercise patients should perform—the key is to encourage them to choose something fun and accessible, so exercise becomes a sustainable part of their life. While exercise can be helpful to prevent migraine, it may not be best for patients to exercise during an acute attack. To date, there is not a lot of evidence suggesting that one diet is more effective at preventing migraine compared to another. However, we do know that skipping meals is a migraine trigger [118]. The patient should aim to eat at least three meals a day and consider adding a morning or afternoon snack to decrease the risk of a migraine occurring between meals. Furthermore, caffeine consumption should be limited to 200 mg of caffeine per day or less [120].

Migraine is a complex neurological condition that in addition to causing pain through widespread sensory activation can also activate the limbic system [30]. For patients, this means that migraine pain will have an emotional component, which can make it difficult to keep track of attacks without a migraine diary. Encouraging patients to keep a diary not only helps to define the frequency and severity of attacks but also helps to determine the effectiveness of treatments and evaluate potential migraine triggers.

#### 3.4.3. Fecal Microbiome Transplantation (FMT)

FMT has been used successfully in the treatment of patients with depression [7,121]. The procedure involves transplanting a healthy fecal microbiome into a person with an unhealthy microbiome. Many patients note the unpleasantness of the procedure as a limitation. Additionally, there is a risk of infection, which can be mitigated through appropriate screening. While several studies have demonstrated the effectiveness of FMT in several chronic conditions, the exact mechanism of action is not fully known. To date, there have been no FMT clinical trials in migraine patients.

## 4. Diet and Migraine

### 4.1. A Bidirectional Relationship between Diet and Migraine: Dietary Factors That Trigger or Subside Migraine

Individual dietary factors (e.g., foods, beverages, and habits) trigger the onset and increased severity of migraine via proposed vascular, neuropeptide, neuroinflammation, insulin, and oxidant–antioxidant pathways [122,123,124]. Common triggers include chocolate, nuts, citrus, cheese, and other dairy products [125,126,127]. Alcohol has also been identified as a common dietary factor associated with migraine, with 17.5% to 35.6% of individuals reporting the beverage as a potential trigger [125,126,127]. When compared to other types of alcoholic beverages, red wine is the most commonly reported trigger by individuals suffering from migraine [127]. Caffeinated beverages, such as coffee, energy drinks, teas, and sodas, are also potential triggers, yet findings are inconsistent. Some studies have demonstrated a positive association between caffeine consumption and migraine onset [128,129]. However, Mostofsky et al. [130] found that one to two servings of caffeine were not associated with migraine, whereas consuming three or more servings demonstrated significantly higher odds of developing a migraine within the same day. With this finding in mind, a daily intake of 200 mg per day of caffeine has been recommended, with the abrupt cessation of caffeine being inadvisable due to withdrawal also being associated with migraine onset [131]. While a healthy balance of caffeine is recommended to prevent migraine, it has also been proposed as a potential treatment due to its initiation of cerebral vasoconstriction. There is insufficient evidence to support universal guidelines for the use of dietary and medicinal caffeine in headache disorders. A sensible approach, based on available evidence, is to limit dietary caffeine intake to moderate amounts with consistent timing before noon and to use caffeine-containing combination analgesics infrequently for milder headaches [132]. One dietary intervention that improves migraine outcomes through the removal of these common migraine triggers from the daily diet is elimination diets [125]. Numerous randomized control trials (RCTs) have demonstrated the positive effects of elimination diets on reducing the frequency and severity of migraine, especially when the removed items are associated with Immunoglobulin G (IgG)-mediated immune responses [133,134,135].

While specific foods and beverages are linked to migraine onset, dietary habits also play a crucial role. For example, fasting due to dietary preferences or religious practices has been found to trigger migraine. During the Holy month of Ramadan, daily fasting caused a significant increase in migraine frequency and severity in practicing Muslims when compared to previous months [136,137,138]. Conversely, a study examining the onset of migraine in 34 individuals following their typical dietary habits over six weeks demonstrated that consuming meals or snacks late at night reduced the odds of experiencing a headache within the consecutive 24 h [139]. The dehydration and hypoglycemia often associated with fasting or extended timeframes between meals are some hypothesized underlying mechanisms for migraine onset due to increased vasopressin secretion, upregulation of the sympathetic nervous system, and alteration of the serotonergic system.

Nutraceuticals have been reported as effective and safe alternatives for individuals with migraine and include vitamins such as B and D; minerals; supplements, such as riboflavin, antioxidants, L-Carnitine, and Omega-3; and phytomedicines such as feverfew (*Tanacetum parthenium*), butterbur (*Petasites hybridus*), cannabis, St. John’s Wort (*Hypericum perforatum*), and ginkgo (*Ginkgo biloba*) [140]. The most commonly used nutraceuticals with evidence for migraine prevention include riboflavin (vitamin B2), coenzyme Q10 (CoQ10), magnesium, butterbur root extract, feverfew, ginkgolide B, and, recently, phycocyanins [141]. While vitamins have strong evidence, dietary therapies offer broader health benefits. With increasing cannabis legalization, providers should note its limited evidence in migraine treatment. Future research should explore traditional medicines and larger human trials for current and new nutraceutical treatments.

Isolated nutrients have also been found to influence migraine outcomes. For example, the increased consumption of B vitamins (e.g., vitamin B6, folate, thiamine, and riboflavin) and magnesium has been shown to reduce or prevent migraine [142]. A study examining the effect of dietary vitamin B6 and folate on migraine outcomes using the National Health and Nutrition Examination Survey found that a vitamin B6 intake of > 2.39 mg per day and a folate intake of > 502.01 mg per day demonstrated significantly lower odds of developing migraine [142]. Similarly, studies have demonstrated an inverse relationship between the increased consumption of dietary thiamine or riboflavin and decreased migraine onset [143,144]. Hypomagnesemia has also been observed in individuals who experience migraine. One cross-sectional study of 905 individuals experiencing migraine demonstrated that they consumed significantly less magnesium (mean = 290.2 mg/day; standard error [SE] = 5.7) when compared to 2721 healthy controls (mean = 327.6 mg/day; SE = 4.7) [145]. Additionally, individuals who attained the recommended dietary allowance of magnesium (310–420 mg/day) through dietary or supplementary methods were at significantly lower odds of migraine onset. While B vitamins play a crucial role in migraine pathogenesis due to their association with mitochondrial energy metabolism [127,143,144], magnesium blocks N-methyl-D-aspartate (NMDA) receptors, which prevents glutamate from binding to the NMDA receptor and causes hyperexcitability in the brain [145]. Dietary changes are one method to improve migraine by increasing the consumption of these essential nutrients. For example, the Mediterranean diet consists of eating primarily legumes, fish, whole grains, olive oil, vegetables, fruits, and nuts, which have high levels of B vitamins and magnesium. Previous studies confirm the effectiveness of this diet, demonstrating significant associations between consuming foods frequently found in the Mediterranean diet and decreased frequency, duration, and severity of migraine [146,147].

Therefore, the relationship between diet and migraine is complex and bidirectional, encompassing how dietary factors can trigger or alleviate migraine and how migraine can influence dietary habits [148]. Identifying and avoiding specific dietary triggers, while incorporating beneficial nutrients, can be a crucial part of migraine management. Migraine can significantly influence an individual’s eating habits. During migraine attacks, symptoms like nausea and vomiting may lead to reduced food intake and the avoidance of certain foods. Post-migraine fatigue can also alter eating patterns, often leading to irregular meal timings and poor nutritional choices. Chronic migraine can lead to long-term changes in dietary habits and nutritional status. Moreover, the stress and pain associated with chronic migraine can lead to emotional eating or appetite suppression, further complicating nutritional status. It is important to state that there is considerable individual variability in how diet affects migraine, influenced by genetic, environmental, and lifestyle factors. The gut microbiome, which plays a key role in digestion and overall health, also varies significantly among individuals and can influence both diet and migraine susceptibility. Understanding these individual differences is therefore essential for personalized dietary recommendations. Behavioral strategies, such as mindful eating and stress management techniques, can also help reduce migraine incidence. Tailoring dietary recommendations to the individual, considering their unique triggers, preferences, and lifestyle, is crucial for effective migraine management.

### 4.2. Dietary Interventions

Certain dietary factors are linked to migraine occurrence and aggravation. A low intake of zinc [149], iron (and low ferritin levels) [150], and potassium [151] is associated with higher migraine prevalence, according to population-based studies. These studies, however, relied on self-reported severe headaches or migraine. The ketogenic diet (KD) is a diet with strong evidence for migraine prevention [152]. The KD, a high-fat, high-protein, low-carbohydrate diet, induces ketosis, which provides alternative energy sources for the brain and reduces cerebral reactivity [153,154]. Studies, including case reports, series, and randomized controlled trials (RCTs), have shown that the KD can significantly reduce migraine frequency, severity, and the need for rescue medication, though it may cause gastrointestinal issues, muscle cramps, fatigue, and increased cholesterol [155]. Since 2021, additional trials have supported the KD’s benefits, showing up to 60% of patients with a ≥50% reduction in monthly headache frequency, even for those with chronic migraine [156]. However, results for low-glycemic-index diets (LGIDs) are mixed [157,158], with some studies finding them less effective than the KD and others finding them equally effective. The optimal diet protocol, including the best lipid/carbohydrate and protein ratios, remains unclear and perhaps continues to do so as the individual needs must be taken into consideration for adjustments and fine-tuning. Neri et al. [159] have systematically reviewed the available literature on migraine and ketosis and recommended the evaluation of ketosis in future interventions. Adverse effects of the KD are generally mild, but long-term adherence is challenging, requiring a multidisciplinary approach [160]. An alternative to the KD is exogenous ketone supplementation to increase ketone bodies in the blood and brain. While this approach is easier to follow than the KD, it has shown no significant clinical improvement over a placebo in recent trials, except for a subgroup of patients with specific metabolic and inflammatory markers [161,162].

Moderate-quality evidence suggests that besides the KD, Dietary Approaches to Stop Hypertension (DASHs) can also reduce the frequency, duration, and severity of migraine in adults [8]. The DASH diet, designed to combat hypertension, emphasizes fruits, vegetables, and whole grains while limiting sodium, sweets, and saturated fats. It has been shown to decrease migraine frequency and severity, reduce headache duration, and lower the Migraine Headache Index Score. Other researched diets include the Mediterranean and MIND diets. The Mediterranean diet, rich in vegetables, legumes, fruits, nuts, olive oil, and limited meat, yields similar benefits comparable to the DASH diet [124,146]. The MIND Diet, created to prevent Alzheimer’s, has shown minimal effects on migraine pain in women [163].

The benefits of omega-3 and omega-6 fatty acids and balancing these acids have been proposed for migraine prevention and treatment [164], where reducing omega-6 and increasing omega-3 intake have been suggested to help reduce migraine attacks [165]. Maintaining stable blood sugar levels seems to benefit individuals with migraine. A 2018 experiment showed that a low-glycemic-index diet decreased attack frequency within the first month of starting the low-glycemic-index diet [166]. Epigenetic diets have been proposed to influence cellular structures and molecules, like mitochondria and DNA, through specific dietary ingredients. This concept, introduced by Hardy and Tollefsboll [167], suggests that dietary components can affect the epigenetic system and potentially prevent diseases, but there are discussions around its applicability [168]. Folate plays a role in DNA methylation, and abnormal mitochondrial DNA methylation has been observed in migraine patients [169]. Future studies may focus on DNA methylation and histone modification, i.e., epigenetic mechanisms influenced by diet. In migraine prevention, folate (vitamin B9) and riboflavin (vitamin B2) are found to be promising compounds [170,171].

Tryptophan-rich foods (e.g., flaxseed, salami, lentils, turkey, nuts, and eggs) may reduce migraine attacks due to their role in serotonin and kynurenine production [172]. Migraine patients often have lower serotonin and tryptophan levels between attacks and higher levels during attacks. Abnormal kynurenine levels have been linked to chronic migraine, suggesting that tryptophan-rich diets might help in prevention and treatment. However, further research is needed as the effects of tryptophan intake have shown mixed results [10].

Various other types of diets have also been tested and interested readers are referred to excellent reviews available, for example, the review by Jibril et al. [173]. Plant-based foods have also attracted attention for their potential beneficial effects on migraine [174,175]. The LIFE diet (Low Inflammatory Foods Everyday), which is a nutrient-dense, dark green leafy vegetable-rich, and whole food plant-based diet, has improved chronic migraine in a case [176].

Researchers worldwide are striving to define “a healthy plate” [177], which is a challenging task considering global differences in resources and practical issues related to sustainability and affordability according to the EAT-Lancet Commission on healthy diets from sustainable food systems [178]. Accordingly, a healthy plate and its relation to migraine have been the subject of various studies and it seems to continue attracting the interest of patients, nutritionists, and headache specialists. For example, an interventional study with a Healthy Eating Plate (HEP) for migraine has been conducted [179], where it was demonstrated that following HEP advice, especially reducing carbs and red and processed meat, migraine frequency and associated disability subsided [179]. This study shows that education and promoting healthy eating habits can be beneficial in migraine.

It is crucial to consider lifestyle factors, age, sex, and individual needs when creating dietary plans or defining a healthy plate for migraine. So far, no certain “migraine diet” exists, and researchers tend to emphasize that dietary recommendation for migraine is a better term to highlight the need for individualization based on patients’ needs and responses to a planned diet. In this line, it seems rational to also take into account diet quality [180] and dietary adherence [181] together with providing evidence for the mechanism-based [182] effectiveness of selected diets, including the potential role of neuroendocrine signaling [183] and the possible contribution of microbiota [184] followed by delineating recommendations for clinical implications [105], for example, the use of pre- or probiotics. Parohan et al. [185] performed a meta-analysis of randomized controlled trials and concluded that probiotic supplementation did not significantly alter the frequency or severity of episodic migraine attacks. However, only three randomized controlled trials (179 patients) were included for analysis and the significant heterogeneity among the studies [185] calls for a cautious interpretation of data and the need for further studies.

Overall, dietary interventions or recommendations may aid in the immediate control, slow progression, or prevention of migraine. Applying a patient-centric model, considering patients’ preferences, comorbidities, and a broader lifestyle modification, including sleep hygiene, stress management, regular exercise, or smoking cessation, seems logical. Accordingly, the effect of migraine or its evolution over age and among the genders on dietary choices must be taken into consideration with dietary patterns, quality, and the amount and the dynamicity of migraine–diet cross-talk [8,69,186,187].

## 5. The Interplay between Diet, Gut Microbiome, and Migraine: Current Evidence

**Figure 1:**
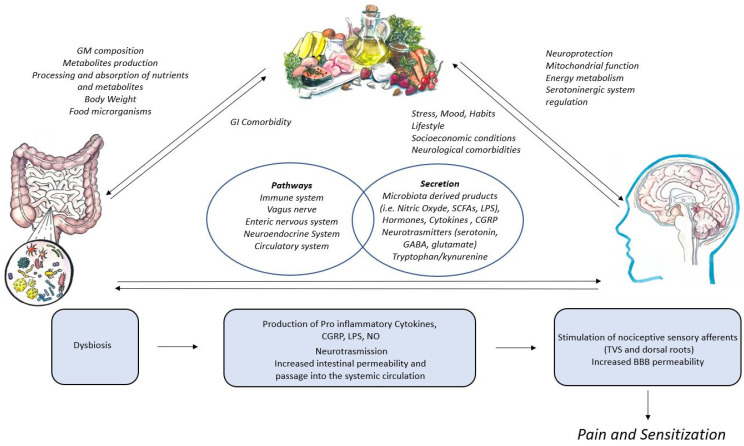
The interplay between diet, gut microbiome, and brain in the pathogenesis of migraine.

While the impact of dietary factors and the gut microbiome on migraine outcomes have been independently examined, limited studies have explored the interplay between these factors via the hypothesized MGB axis. One RCT examining the influence of symbiotic supplementation on gut microbial by-products and migraine outcomes in women demonstrated a reduction in the frequency of migraine and the use of painkillers in individuals who received the symbiotic treatment [188]. Gut microbial by-products, specifically C-reactive protein and zonulin, also decreased in the treatment group, suggesting a reduction in inflammation and intestinal permeability. However, the direct influence of symbiotic supplementation on the diversity and composition of the gut microbiome was not explored. A murine model investigated how the gut microbiome’s composition and nitroglycerin-induced migraine are influenced by sodium butyrate (SB) and sodium propionate (SP), two SCFAs that are found in the diet [189]. SB and SP administration was associated with a higher Firmicutes-to-Bacteroidetes ratio, demonstrating an increase in healthy bacteria commonly found in the gut microbiome of mice and humans. High levels of SB (30 mg/kg) and SP (100 mg/kg) treatments reduced pain following the injection of nitroglycerin, which was used to induce migraine-like symptoms. Another RCT that examined how the adoption of a gluten-free Mediterranean diet influenced the gut microbiome and migraine outcomes corroborated the influence of dietary factors on migraine outcomes in humans diagnosed with gastrointestinal disorders [190]. Individuals who maintained a gluten-free diet demonstrated a significant reduction in migraine frequency over the previous three months. While there were no significant differences in bacterial and fungal diversity (alpha and beta) within the gut microbiome when comparing the gluten-free participants to controls (receiving 8 g of gluten daily), the gluten-free participants demonstrated a significant reduction in the abundance of the fungus Dothideomycetes, whereas the controls experienced a decrease in the fungus Tremellomycetes. These preliminary results highlight the importance of future research examining the interplay between dietary factors, the gut microbiome, and migraine via the proposed MGB axis to develop a deeper understanding of the pathogenesis of migraine. The insights that derive from this future work can foster the development of novel migraine dietary treatments or potentially provide a biomarker from which targeted and individualized dietary interventions can be formulated [191]. Figure 1 depicts this proposed interplay.

Different dietary components can affect the composition of the gut microbiome. The diversity of the gut microbiome, its ecosystem, and the production of metabolites are influenced by the metabolism of nutrients entering the intestine from the diet. The microbiome–gut–brain axis is interconnected by the immune system, vagus nerve, enteric nervous system, neuroendocrine system, and metabolomic system. This communication occurs through sensory nervous pathways and also involves the release of numerous gut microbiome products into the systemic circulation. Similarly, the brain can communicate with the intestine directly by releasing neuroactive substances or changing the gut microbiome. The interaction between the gut microbiome, brain, and diet is not one-way but rather multidirectional. The intake of specific foods can be impacted by gastrointestinal disorders. Diet has a direct impact on brain functions through mechanisms that include neuroprotection, energy metabolism, and the regulation of mitochondrial and serotonergic functions. Finally, neurological or neuropsychiatric conditions, lifestyle, and socioeconomic factors can be important determinants of eating habits. Dysbiosis at the intestinal level could be favored by certain dietary habits, which alter the production of metabolites and establish a pro-inflammatory biochemical structure in the intestine. Chemical stimuli possess the ability to directly stimulate sensory fibers in the gut, which are then able to transmit messages to the brain. Furthermore, the proinflammatory environment can lead to an increase in intestinal permeability and passage into the systemic circulation of inflammatory molecules, which can stimulate the trigeminovascular system and spinal sensory afferents with the facilitation of the triggering of pain and also sensitization phenomena.

## 6. Conclusions

This review has elucidated the intricate and multidirectional relationship between diet, the gut microbiome, and migraine. Key findings highlight that dietary habits and gut health significantly influence migraine pathogenesis, while migraine can alter eating behaviors and nutritional status. The literature provides evidence that certain foods and beverages can act as migraine triggers, while nutrients like magnesium and riboflavin may help reduce migraine frequency and severity. The composition of the gut microbiome plays a crucial role in modulating migraine susceptibility through mechanisms, such as neuroinflammation, gut–brain signaling, and metabolic function. Migraine can also lead to altered eating patterns, including reduced food intake during attacks and poor nutritional choices, which can exacerbate the condition and affect overall health. There is significant individual variability in how diet affects migraine, influenced by genetic, environmental, and lifestyle factors, including the unique composition of each individual’s gut microbiome. Therefore, personalized dietary interventions, such as elimination diets and the inclusion of beneficial nutrients, show promise in managing migraine symptoms and improving quality of life. Advanced digital tools can aid in continuous monitoring, offer tailored educational content, support adherence to dietary plans, and enhance personalized care. Considering the complex interactions of lifestyle factors, as well as the influence of age and sex, is essential in creating effective dietary plans that meet patients’ needs at different life stages. The relationship between diet and migraine is dynamic and bidirectional, requiring careful monitoring and individualized dietary choices to achieve the best outcomes and minimize potential risks.

This review offers implications for future research and clinical practice. Future research should focus on large-scale, long-term studies to better understand the mechanisms underlying the diet–gut–migraine relationship and identify precise dietary recommendations. Advances in understanding individual variability in diet and gut microbiome composition will pave the way for personalized migraine management strategies, tailored to the unique needs of each patient. From this perspective, it should be considered that the microbiome is a true metabolic system made up not only of microorganisms but also of their metabolites. Metabolomic and metaproteomic studies can help understand which dietary styles in migraineurs can create a more beneficial microbiome ecosystem.

Developing evidence-based clinical guidelines for dietary and lifestyle interventions in migraine management will be crucial for healthcare providers, offering clear and actionable recommendations for patients. Integrating dietary management, gut health optimization, and lifestyle modifications into a holistic approach to migraine treatment can potentially reduce the frequency and severity of migraine, improving patient outcomes and quality of life. By continuing to explore the complex interactions between gut microbiome and dietary choices and their implications, we can move towards more effective and individualized approaches to managing migraine, ultimately reducing the burden of this debilitating condition.
